# Central core disease

**DOI:** 10.1186/1750-1172-2-25

**Published:** 2007-05-15

**Authors:** Heinz Jungbluth

**Affiliations:** 1 Evelina Children's Hospital, Department of Paediatric Neurology, St. Thomas' Hospital, London, UK

## Abstract

Central core disease (CCD) is an inherited neuromuscular disorder characterised by central cores on muscle biopsy and clinical features of a congenital myopathy. Prevalence is unknown but the condition is probably more common than other congenital myopathies. CCD typically presents in infancy with hypotonia and motor developmental delay and is characterized by predominantly proximal weakness pronounced in the hip girdle; orthopaedic complications are common and malignant hyperthermia susceptibility (MHS) is a frequent complication. CCD and MHS are allelic conditions both due to (predominantly dominant) mutations in the skeletal muscle ryanodine receptor (*RYR1*) gene, encoding the principal skeletal muscle sarcoplasmic reticulum calcium release channel (RyR1). Altered excitability and/or changes in calcium homeostasis within muscle cells due to mutation-induced conformational changes of the RyR protein are considered the main pathogenetic mechanism(s). The diagnosis of CCD is based on the presence of suggestive clinical features and central cores on muscle biopsy; muscle MRI may show a characteristic pattern of selective muscle involvement and aid the diagnosis in cases with equivocal histopathological findings. Mutational analysis of the *RYR1 *gene may provide genetic confirmation of the diagnosis. Management is mainly supportive and has to anticipate susceptibility to potentially life-threatening reactions to general anaesthesia. Further evaluation of the underlying molecular mechanisms may provide the basis for future rational pharmacological treatment. In the majority of patients, weakness is static or only slowly progressive, with a favourable long-term outcome.

## Disease name

Central core disease

## Definition

Central core disease (CCD) (MIM * 117000) [1] is an inherited neuromuscular disorder defined by a) areas with reduced oxidative activity running along the longitudinal axis of the muscle fibre ("central cores") and b) clinical features of a congenital myopathy.

CCD was originally reported in a family with congenital hypotonia, non-progressive weakness and central areas of amorphous appearance within muscle fibres stained with the modified Gomori trichrome technique [2]. The term CCD was introduced later [3] and reflects the characteristic absence of oxidative enzyme activity in the core area due to mitochondrial depletion [4].

## Epidemiology

Epidemiological data are only available for the congenital myopathies as a group but not for specific conditions. The incidence of all congenital myopathies is estimated at around 6.0/100,000 live births, or onetenth of all cases of neuromuscular disorders [5]. Regional studies in Northern Ireland [6] and Western Sweden [7] suggest a prevalence between 3.5 – 5.0/100,000 in a paediatric population.

CCD is probably the most common congenital myopathy; the condition is likely to be under recognised considering that some individuals with suggestive clinical features and an identical genetic background do not necessarily exhibit the characteristic histopathological features, particularly when biopsied at an early age.

## Clinical description

Presentation of dominantly inherited CCD is typically in infancy with hypotonia or in early childhood with motor developmental delay [8]; however, marked clinical variability, often within the same family, has been recognised [9-12]. Muscle stiffness and weakness on exertion are recognised presenting features [13,14]. More severe presentations within the range of the foetal akinesia sequence [15] have been reported associated with recessive inheritance. There is no association between the number of cores on muscle biopsy and the degree of muscle weakness [16].

Distribution of weakness is typically proximal with prominent involvement of the hip girdle and also axial muscles [8]; rare patients may show focal wasting [17]. Inability to bury eyelashes completely may be the only manifestation of typically mild facial involvement. Bulbar involvement is untypical in the dominant form and extra-ocular muscle involvement has been considered a clinical exclusion criterion by some authors [18]; however, both features may be observed in the most severely affected neonates due to recessive inheritance [15].

Orthopaedic complications are common in CCD [19] and comprise congenital dislocation of the hips [20], scoliosis which may be present from birth [12] and foot deformities including talipes equinovarus and pes planus [19]. Many affected individuals have marked ligamentous laxity, occasionally associated with patellar instability [19], whereas contractures other than tendon Achilles tightness are rare.

Structural cardiac abnormalities other than mitral valve prolapse have rarely been reported [13], but cardiomyopathies are not a feature of CCD associated with *RYR1 *mutations. Central cores on muscle biopsy have been observed in a group of patients with hypertrophic cardiomyopathy secondary to mutations in the beta-myosin heavy chain (*MYH7*) gene, however, these patients typically do not have associated muscle weakness or any other features of typical CCD [21]. Central and minicores in association with a dilated cardiomyopathy may also rarely been observed in patients with mutations in the skeletal muscle **α**-actin (*ACTA1*) gene [22], more frequently associated with nemaline myopathy.

Respiratory involvement in dominant CCD is exceptional but may be severe in neonatal cases due to recessive *RYR1 *mutations [15].

Malignant hyperthermia, a pharmacogenetic disorder of skeletal muscle characterised by an abnormal response to muscle relaxants such as succinylcholine and volatile anaesthetics, is a frequent complication [23-25]. Malignant hyperthermia is a severe and occasionally fatal reaction characterised by muscular rigidity, rhabdomyolysis, rapid increase in body temperature and signs of generalised metabolic decompensation; survivors may suffer severe renal and neurologic damage. Many patients with CCD test positive for the malignant hyperthermia susceptibility (MHS) trait on *in vitro *contracture test (IVCT) [13,26] and should therefore be considered at risk for malignant hyperthermia during general anaesthesia.

Almost all patients with CCD achieve the ability to walk independently, except the most severe neonatal cases and some of those with congenital dislocation of the hips [27,28]. CCD typically follows a static or only slowly progressive course, even over prolonged periods of follow-up [29]. Intermittent deterioration of symptoms has been reported during or after pregnancy [30].

Serum creatine kinase (CK) activity is usually normal, but may be elevated up to 6 to 14 times normal in rare cases [11,31,32]. Muscle ultrasound often shows a striking increase in echogenicity even in paucisymptomatic individuals [33]. A characteristic pattern of selective involvement on muscle magnetic resonance imaging (MRI) has been reported in patients with typical CCD [34] and is distinct from that observed in other congenital myopathies such as nemaline myopathy [35]; muscle MRI may therefore be particularly useful for aiding genetic diagnosis in cases with mixed pathologies featuring both cores and rods [36,37].

## Aetiology

CCD is due to mutations in the skeletal muscle ryanodine receptor (*RYR1*) gene at chromosome 19q13.1, also implicated in the malignant hyperthermia susceptibility (MHS) trait, initially recognised as a familial autosomal dominant trait by Denborough and Lovell [23] in Australia.

An association between CCD and MHS had been suspected early, as individuals with MHS may have central cores on muscle biopsy [38], and patients with CCD may be prone to malignant hyperthermia episodes [13,39,40]. The *RYR1 *gene had been considered as a candidate for malignant hyperthermia based on the finding of a founder mutation in the porcine isoform resulting in the porcine stress syndrome (PSS), a naturally occurring animal model with almost identical clinical features [41]. Following demonstration of linkage to the human *RYR1 *locus in some families with MHS [42-45] and CCD [46-48], subsequent identification of mutations in the *RYR1 *gene suggested that the two conditions are allelic disorders [49-51].

*RYR1 *is organized in 106 exons [52] and encodes the skeletal muscle ryanodine receptor (RyR1), a large protein of 5037 amino acids visible on electron microscopy. RyR1 is a ligand-gated release channel for Ca^++ ^stored in the terminal cisterna with a crucial role in excitation-contraction (E-C) coupling by regulating cytosolic calcium levels. RyR1 calcium release is primarily triggered by voltage-induced conformational changes of the abutting dihydropyridine receptor (DHPR), and secondarily by a number of exogeneous and endogeneous effector molecules (for review, [53]). RyR1 N-terminal portions are myoplasmic and constitute the visible foot structure that interacts with the DHPR, whereas the actual calcium release channel is located in the C-terminal part of the protein [54,55].

More than 80 mutations have been identified in the *RYR1 *gene to date [56-63], most of them missense mutations. A few small deletions [59,60,64] and cryptic splicing site mutations [63,65] have been documented. The majority of *RYR1 *mutations associated with MHS or CCD described to date were dominant mutations; homozygosity or heterozygosity for *RYR1 *mutations has been previously documented in association with MHS [66,67] and have been recently reported in a severe form of CCD presenting with a foetal akinesia syndrome [15] and few mild cases [16].

Genotype-phenotype correlations associated with mutations in the *RYR1 *gene are complex and may be partly explained by the degree of functional differentiation within this large protein. Dominant *RYR1 *mutations affecting the cytoplasmic N-terminal (MHS/CCD region 1, amino acids 35 – 614) and central (MHS/CCD region 2, amino acids 2163 – 2458) domains of the protein give predominantly rise to the MHS phenotype [68], whereas the CCD phenotype is closely associated with dominant *RYR1 *C-terminal (MHS/CCD region 3, amino acids 4550 – 4940) mutations [16,58-61]. CCD-related dominant *RYR1 *mutations affecting the N-terminal and central portions of the protein and *RYR1 *C-terminal mutations giving rise to MHS represent exceptions to this rule but have only been reported in a few families [50,51,69-71]. Recessively inherited mutations are more widespread throughout the *RYR1 *gene and appear to be more frequently associated with the histopathological appearance of Multi-minicore Disease (MmD) [27,63-65,72] rather than CCD [16]. Data regarding the frequency of CCD-related *RYR1 *mutations are currently emerging.

Although many mutations are private, the Arg4861Cys substitution has been identified in three unrelated CCD families and mutations affecting *RYR1 *exons 100 – 101 appear to be particularly common [16,61,62]. Since most previous genetic studies in MHS and CCD were limited to a partial screening strategy due to the large size of the *RYR1 *gene, future studies involving the entire *RYR1 *coding sequence are likely to identify new mutational hotspots.

Mutation-induced conformational changes of the RyR1 protein are thought to alter excitability and/or calcium homeostasis within muscle cells, but the precise molecular mechanisms underlying genotype-phenotype correlations associated with specific *RYR1 *mutations are currently still emerging. Two models for receptor malfunction have been proposed, depletion of sarcoplasmic reticulum calcium stores with resulting increase in cytosolic calcium levels ("leaky channel" hypothesis) [73], and disturbance of excitation-contraction coupling (E-C uncoupling hypothesis) [74]; these models are not mutually exclusive and may be equally valid depending on the specific effect of individual mutations on the structurally complex RyR1 protein.

The functional effects of specific *RYR1 *mutations have been studied in response to IVCT, in cultured myotubes from patients and in various homologous and heterologous expression systems. Early studies already suggested an association between specific *RYR1 *mutations and IVCT response [75], and indicated a correlation between the particular *RYR1 *gene mutation in a family and the severity of MH or liability to CCD [26,76]. *In vitro *studies on human myotubes demonstrated both increased [77,78] and reduced [79,80] agonist sensitivity depending on the precise location of the *RYR1 *mutation investigated. Studies on a *RYR1 *C-terminus mutation (I4898T) associated with a severe CCD phenotype demonstrated reduced intracellular calcium release without increasing the sensitivity to caffeine or halothane [36] and a marked disturbance of excitation-contraction coupling [81]; the latter may also apply for other C-terminal mutations [82]. Studies of *RYR1 *mutants expressed in myotubes of *RYR1 *knockout ("dyspedic") mice [83,84] indicate that mixed MHS/CCD mutations are associated with increased channel activity sufficient to deplete sarcoplasmic reticulum calcium stores, elevated intracellular calcium levels and reduced maximum voltage gated calcium release [79], whereas MH-only mutations appear to increase basal release channel activity insufficiently to alter net sarcoplasmic reticulum calcium content ("compensated leak"). Expression of a functional RyR1 protein in B-lymphocytes has been recently described and offers a novel approach to study the pathogenesis of *RYR1 *mutations *in vitro*. B-lymphocytes harbouring CCD-related *RYR1 *mutations show depletion of sarcoplasmic reticulum stores secondary to unprompted calcium release [59,85]; increased release of inflammatory cytokines in the same study population [86] may also point at a role of *RYR1 *in immunomodulation.

Although most studies have confirmed the association between CCD and mutations in the *RYR1 *gene, possible genetic heterogeneity is indicated by recombination events between CCD and *RYR1 *in one family [87], and failure to identify a mutation despite screening of the entire *RYR1 *coding sequence in a severely affected neonate with histopathologic and clinical features of CCD [15].

## Diagnostic methods

The diagnosis of CCD depends on the presence of typical histopathological findings on muscle biopsy in combination with suggestive clinical features; muscle MR imaging may complement clinical assessment.

**Figure 1:**
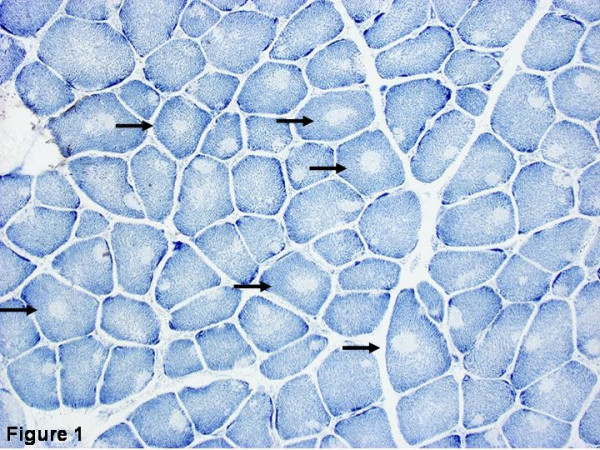
**Histopathologic appearance of typical central core disease**: NADH-TR, transverse section from the rectus femoris. Marked predominance of dark staining, high oxidative type 1 fibres with cores affecting the majority of fibres. Cores are typically well demarcated and centrally located (→), but may occasionally be multiple and of eccentric location.

Histochemical staining of **muscle biopsy **samples shows the absence of oxidative and glycolytic enzymatic activity from central core regions [4] (Figure 1). Central cores may be single or multiple, central or eccentric [88,89], but commonly run along a significant extent of the longitudinal muscle fiber axis [90,91]; the latter feature distinguishes central cores most clearly from multi- or minicores which share focal reduction of oxidative activity as a common histopathological feature. Central cores are typically found in type 1 fibres; marked type 1 predominance or uniformity and hypotrophy are typical and may be the only abnormal features at presentation [89,92]. An increase in internal nucleation is often associated and substantial increases in fat and connective tissue have been described in some patients [89], corresponding to a marked degree of increased signal intensity on muscle MRI [35]. The degree of histopathologic changes may be variable depending on sampling site and the age of the patient.

Typical findings on electron microscopy include reduction or absence of mitochondria, variable degrees of myofibrillar disorganisation and accumulation of abnormal Z band material within the usually sharply demarcated core area [9]. Some ("structured") cores preserve a degree of myofibrillar organisation and therefore retain some ATPase activity; other ("unstructured") cores do not and can be found in the same muscle biopsy [32,90]. The sarcoplasmic reticulum and Ttubule structures may be increased within the core area [93].

Abnormal expression of various sarcomeric and intermediate filament proteins, particularly desmin, has been demonstrated within or around the core-area in a number of immunohistochemical studies; corresponding to type 1 predominance on histochemical stains, slow myosin isoforms may be upregulated [89,94-96]. An antibody to the actin cross-linking protein filamin C has been recently identified as a strong but non-specific marker of central cores [97].

**Figure 2:**
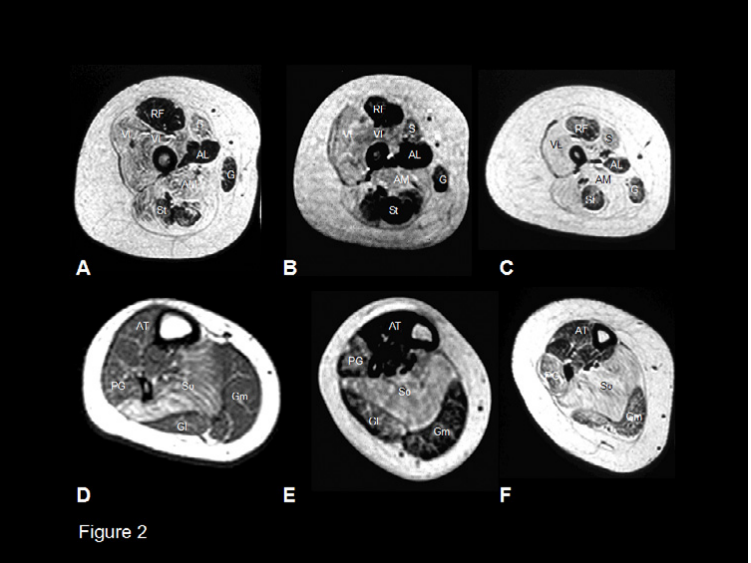
* Muscle involvement in the lower limbs in CCD secondary to dominant *RYR1 *mutations: T1-weighted MR imaging, transverse sections of the proximal thigh (A-C) and the proximal lower leg (D-F) in an eleven (A) and a thirteen year old boy (B,E), and a twelve (C,F) and seventeen year old girl (D). In the thigh (A-C), there is marked increase in abnormal signal within vasti, sartorius and adductor magnus with relative sparing of rectus femoris, adductor longus, gracilis and semitendinosus. In the lower leg, there is increase in abnormal signal in soleus (D-F), and – in more severe cases (E-F) – peroneal group and gastrocnemius medialis. Tibialis anterior and gastrocnemius lateralis are relatively spared. (VL = vastus lateralis, VI = vastus intermedius, VM = vastus medialis, RF = rectus femoris, AL = adductor longus, AM = adductor magnus, S = sartorius, G = gracilis, St = semitendinosus). * Reprinted from *Neuromuscul Disord *2004, **14: **Jungbluth H, Davis MR, Muller C, Counsell S, Allsop J, Chattopadhyay A, Messina S, Mercuri E, Laing NG, Sewry CA, Bydder G, Muntoni F. **Magnetic resonance imaging of muscle in congenital myopathies associated with RYR1 mutations**. Pages: 785–790. Copyright Owner Elsevier, Copyright (2004), with permission from Elsevier".

In cases with equivocal histopathological features, **muscle MR imaging **may complement clinical assessment and indicate involvement of the *RYR1 *gene, as *RYR1 *C-terminal mutations may be associated with a consistent pattern of selective muscle involvement [35] (Figure 2) distinct from that observed in other congenital myopathies such as nemaline myopathy [34].

## Differential diagnosis

The diagnosis of CCD is usually straightforward in cases where clinical features are suggestive and typical central cores are present on muscle biopsy; however, the typical histopathological picture may only evolve over time and may not always be present when muscle biopsy has been performed at an early age. Also, core-formation is a non-specific finding which may be observed in other clinical contexts such as tenotomy, denervation ("target-fibers") [98] or malignant hyperthermia susceptible individuals without other features of a congenital myopathy [99]; it has to be emphasised that the presence of central cores on muscle biopsy without associated weakness is not sufficient to constitute a diagnosis of CCD.

The distinction from **Multi-minicore Disease (MmD) **may be particularly difficult, as recent studies suggest a histopathological continuum between the two conditions in a subset of patients rather than distinct entities. Although the great majority of MmD is caused by recessive mutations in the selenoprotein N (*SEPN1*) gene [100], recessive *RYR1 *mutations have been recently identified in distinct subgroups of MmD [27,63,65,72]. However, MmD-associated mutations give rise to clinical features such as external ophthalmoplegia, bulbar involvement and a moderate degree of respiratory impairment which are not commonly observed in typical CCD.

**Other conditions which may feature central cores on muscle biopsy **include hypertrophic cardiomyopathy (HCM) associated with missense mutations in the **β**-myosin heavychain gene, *MYH7 *[21]; in contrast to CCD, muscle weakness is, however, exceptional and there are no associated musculoskeletal deformities. Mutations in the *ACTA1 *gene, more commonly associated with nemaline myopathy, have been recently implicated in an autosomal dominant myopathy with both central and minicores [22]; however, some patients in this pedigree had an associated cardiomyopathy, which is not a feature in *RYR1*-related CCD.

The common occurrence of central cores and features of other congenital myopathies such as nemaline rods or minicores has been recognised for a long time, poses a diagnostic challenge [37,101-103] and emphasises that histopathological interpretation has to take into account the clinical context.

## Genetic counselling

Genetic counselling should be offered to all families and individuals in whom a diagnosis of CCD has been made. Although the majority of cases appear to be associated with dominant inheritance, care should be taken in interpreting the mode of inheritance in isolated cases, as both sporadic occurrence and, more recently, recessive inheritance [16,27,72] have been documented; an unusually severe presentation with antenatal onset in particular should raise the possibility of a recessively inherited form of CCD.

Molecular genetic confirmation of the diagnosis is possible by mutational analysis of the *RYR1 *gene; diagnostic *RYR1 *screening has been established for malignant hyperthermia patients by a number of laboratories associated with the European Malignant Hyperthermia Group (EMHG) [104] but does require a modified approach for patients with central core disease as many CCD-related *RYR1 *mutations do not localise to known MH hotspots. Technical difficulties associated with the large size of this gene may be partly alleviated by focusing on the C-terminal mutational hotspot [16,61,62], particularly exons 100 and 101, closely associated with the typical dominant form of CCD; however, recessive inheritance due to compound heterozygosity for two different *RYR1 *mutations can not be excluded if only a partial screening strategy is applied and sequencing of the entire *RYR1 *coding sequence may be required in patients without confirmed hotspot mutation.

## Management

No curative treatment is currently available for CCD and management is essentially supportive based on a multidisciplinary approach.

Regular physiotherapy is aimed at the preservation of muscle power and function and the prevention of contractures, particularly those of the tendon Achilles which are commonly observed in CCD. Considering often prominent axial involvement, exercises promoting endurance and truncal stability such as swimming and riding [105] may be particularly useful. Considering a tendency to exercise-induced myalgia in CCD, exercises involving a high-resistance load have to be approached with caution and are probably not recommendable. If complications such as congenital dislocation of the hips (CDH), talipes equinovarus or scoliosis are present at birth or evolve in the course of the disease, those may be managed surgically at a centre with experience in the management of neuromuscular disorders once conservative approaches have failed. As in other neuromuscular conditions, post-operative mobilization ought to be rapid in order to avoid adverse effects of prolonged immobilization such as muscle atrophy. In the most severe cases where walking can not be achieved without additional support, independent ambulation may be promoted by appropriate rehabilitative measures such as provision of weight bearing calipers.

Clinically significant respiratory involvement is exceptional in typical cases of dominant CCD; however, considering the small risk of respiratory impairment, we would advocate regular monitoring of respiratory capacity and annual overnight oxygen saturation studies if forced vital capacity (FVC) is less than 60% of the expected value, and more frequently if FVC is less than 40% [106]. Respiratory infections should be treated actively. In the most severely affected, often recessive CCD cases with antenatal onset, respiratory involvement may be severe enough to require invasive ventilation [15,65]. Although in some of these infants respiratory impairment may be life-limiting, others appear to stabilise or even improve after a period of ventilation; active treatment decision therefore ought to be made on an individual basis applying the same criteria as for other children with neonatally severe conditions.

An associated cardiomyopathy has not been reported in typical CCD due to mutations in the *RYR1 *gene; however, cardiomyopathies associated with mutations in the *MYH7 *[21] and the *ACTA1 *[22] genes may feature central cores on muscle biopsy but do not share the typical clinical features of CCD. Cardiac ultrasound studies therefore ought to be considered in cases where clinical presentation is unusual.

Patients with CCD are at risk of malignant hyperthermia, an abnormal response to muscle relaxants such as succinylcholine and volatile anaesthetics [24,25]. The anaesthetist ought to be aware of the diagnosis of CCD and plan the anaesthesia accordingly, avoiding potentially MH-triggering agents. Clinically manifest MH reactions may be aborted by the RyR1 agonist Dantrolene if administered early in the course of a reaction. Not all patients with CCD will be at risk of MH [36,72] and ideally the MHS status ought to be determined by IVCT testing in individual cases; however, this may not always be practical as the IVCT is not offered for young children and involves an open muscle biopsy under general anaesthesia. It may be more appropriate to assume MH susceptibility in CCD patients in the absence of firm evidence to the contrary and discuss the issue with affected individuals and their families accordingly.

In affected females, the potential for intermittent deterioration of symptoms during or after pregnancy [30] ought to be anticipated and discussed with the patient.

In addition to supportive management and prevention of malignant hyperthermia reactions during general anaesthesia, the **β**-agonist salbutamol has been recently investigated as a pharmacological agent in the treatment of CCD with encouraging results [107]. However, results of this pilot study will have to be validated in a larger randomized controlled trial as a basis for future recommendation.

## Prognosis

The typical form of dominantly inherited CCD is usually associated with a mild to moderate degree of disability and carries an overall favourable prognosis, although the degree of severity may be variable, occasionally within the same family. Apart from the most severe cases and some of those with congenital orthopaedic complications [27,28] almost all patients achieve the ability to walk independently. The course of CCD is static or only slowly progressive, even over prolonged periods of follow-up [29].

In the most severely affected, often recessive CCD cases with antenatal onset, respiratory impairment may be life-limiting despite active management, although others appear to stabilise or even improve after a period of ventilation [65].

## Unresolved questions

CCD and the MHS trait are closely associated with mutations in the *RYR1 *gene, but clear genotype-phenotype correlations are still emerging. Screening of the entire *RYR1 *coding sequence in an increasing number of patients may identify new mutational hotspots in future, and alleviate the difficulties associated with the large size of this gene by a more rationalised screening strategy.

Although the pathogenetic basis of CCD and the malignant hyperthermia susceptibility trait is currently only partially understood, available experimental evidence suggests a mutation-specific disruption of excitation-contraction-coupling and/or disturbance of intracellular calcium homeostasis. Future work is likely to advance our understanding of these key processes in contractile cells, and to elucidate mutation-specific effects resulting in congenital myopathy phenotypes and/or malignant hyperthermia. Further understanding of the molecular basis of CCD and MHS may also provide the basis for rational pharmacological treatments. Unresolved questions concern the pathogenesis of central cores, the impact of specific mutations on RyR1 assembly, the precise role of RyR1 in non-muscle cells such as B-lymphocytes and the effect of mutations on those tissues, and other calcium dependent signalling pathways other than E-C coupling.
